# Exploring Effects of Mental Stress with Data Augmentation and Classification Using fNIRS

**DOI:** 10.3390/s25020428

**Published:** 2025-01-13

**Authors:** M. N. Afzal Khan, Nada Zahour, Usman Tariq, Ghinwa Masri, Ismat F. Almadani, Hasan Al-Nashah

**Affiliations:** 1 Department of Electrical Engineering, American University of Sharjah, Sharjah 26666, United Arab Emirates; khanm@aus.edu (M.N.A.K.); g00096513@aus.edu (N.Z.); utariq@aus.edu (U.T.); g00094347@aus.edu (G.M.); b00096723@aus.edu (I.F.A.); 2 Biosciences and Bioengineering Graduate Program, American University of Sharjah, Sharjah 26666, United Arab Emirates

**Keywords:** functional near-infrared spectroscopy (fNIRS), hemodynamic response, deep convolutional generative adversarial network (DCGAN), feed-forward neural network, linear support vector machines, decision tree, restricted Boltzmann machine, convolutional neural networks, classification, binaural beats

## Abstract

Accurately identifying and discriminating between different brain states is a major emphasis of functional brain imaging research. Various machine learning techniques play an important role in this regard. However, when working with a small number of study participants, the lack of sufficient data and achieving meaningful classification results remain a challenge. In this study, we employ a classification strategy to explore stress and its impact on spatial activation patterns and brain connectivity caused by the Stroop color–word task (SCWT). To improve our results and increase our dataset, we use data augmentation with a deep convolutional generative adversarial network (DCGAN). The study is carried out at two separate times of day (morning and evening) and involves 21 healthy participants. Additionally, we introduce binaural beats (BBs) stimulation to investigate its potential for stress reduction. The morning session includes a control phase with 10 SCWT trials, whereas the afternoon session is divided into three phases: stress, mitigation (with 16 Hz BB stimulation), and post-mitigation, each with 10 SCWT trials. For a comprehensive evaluation, the acquired fNIRS data are classified using a variety of machine-learning approaches. Linear discriminant analysis (LDA) showed a maximum accuracy of 60%, whereas non-augmented data classified by a convolutional neural network (CNN) provided the highest classification accuracy of 73%. Notably, after augmenting the data with DCGAN, the classification accuracy increases dramatically to 96%. In the time series data, statistically significant differences were noticed in the data before and after BB stimulation, which showed an improvement in the brain state, in line with the classification results. These findings illustrate the ability to detect changes in brain states with high accuracy using fNIRS, underline the need for larger datasets, and demonstrate that data augmentation can significantly help when data are scarce in the case of brain signals.

## 1. Introduction

Originally developed for clinical tissue oxygenation monitoring [1], functional near-infrared spectroscopy (fNIRS) has evolved into a useful tool for functional neuroimaging studies [2,3,4]. To date, various fNIRS devices that track changes in local cerebral oxygenation by measuring variations in the concentration of deoxygenated hemoglobin (ΔHbR) and oxygenated hemoglobin (ΔHbO) have been developed. fNIRS has been used to investigate a variety of brain activity, such as cognitive and motor processes [5,6,7,8].

fNIRS is being used more often in functional neuroimaging studies due to its affordability and portability [9]. As opposed to functional magnetic resonance imaging (fMRI), fNIRS measures alterations in the brain’s hemodynamics. It shares similarities with fMRI but stands out for being quiet (with no operating sound). Higher temporal resolution, freedom from space restriction, and no need for participants to lie down are all very appealing features. These characteristics make fNIRS the preferred option for tracking hemodynamic changes associated with brain activity, not only in lab settings but also in more ecologically valid and real-world workspace environments.

As a useful neuroimaging tool, fNIRS has impressive potential and notable applications across multiple domains. Compared with electroencephalography, it has the advantage of having better spatial resolution and being less susceptible to noise [10]. Some example applications of fNIRS include assessing early neurodevelopment [11], cognitive discernment and perception [12], psychiatric conditions [13], language experiment research [14], as well as addressing issues related to stroke and brain damage [15]. Additionally, fNIRS plays a crucial role in clinical and network imaging [16], Brain-Computer Interfaces (BCIs) [17], and mental stress analysis [18,19].

Among the main sources of stress in an individual’s life is their workplace. Stress-related problems can arise when workers are overworked and are unable to fulfill unreasonable deadlines (. Many factors, such as disturbed sleep patterns, excruciating headaches, decreased concentration, and elevated absenteeism, are caused by stress and influence workers. Firms suffer large financial losses because of these ramifications. Anxiety, insomnia, recurrent injuries, and a rise in absenteeism—especially at work—are all associated with stress [20].

A multitude of studies have employed fNIRS as a primary modality for studying cortical activity with different stressors and cerebral regions. A 2018 study that examined the impact of mental stress on occupational performance used multiple virtual training instances and found significant prefrontal cortex activation (PFC) [21]. The increased activity of the PFC was then used to detect stress. The next year, the effects of psychosocial stress on cognition were examined using the Trier Social Stress Test (TSST) [22]. The results showed that, in male teenagers exposed to psychosocial stress, maintaining an increased level of physical activity did not always translate into improved inhibitory control. Additionally, mental arithmetic exercises were employed as stressors in other research projects by different investigators, confirming the efficacy of fNIRS as a tool for early identification and measurement of mental stress [23,24]. The various application of fNIRS across multiple stress paradigms highlights its efficacy as a neuroimaging tool for assessing and quantifying mental stress.

The need for advanced methodologies that can identify and track particular brain states has grown as a result of recent advancements in fNIRS technology, particularly in the field of brain state monitoring for biomedical applications. These applications address issues like stress and chronic diseases like dementia and mild cognitive impairment. One notable issue that arises when incorporating brain images—such as activation maps and connectivity maps—into the training and assessment of modern machine learning algorithms is that there are not enough data, which makes the models more prone to overfitting. Adopting data augmentation techniques becomes essential to lessening this challenge. Conventional augmentation techniques, such as cropping, rotating, and zooming, are frequently utilized; however, they have drawbacks, such as requiring manual intervention and being impractical for complex images such as brain activation maps [25,26].

Modern machine learning techniques, such as Generative Adversarial Networks (GAN), provide a promising alternative to traditional augmentation methods [27]. In the context of brain imaging and machine learning applications, GANs offer a workable solution to the data scarcity dilemma by enabling the creation of realistic augmented data. Typically, conventional GAN models need a significant amount of data to be trained and used for image augmentation. However, recent noteworthy works by Toutouh et al. [28] and Zhao et al. [29] have paved the way for training GAN models even with a limited dataset size, such as a thousand images. These discoveries have greatly helped expand the use of GANs in situations where data availability is limited. Hence, the idea behind enhancing fNIRS data for reliable machine learning model development. This combination of old and new methods, along with improvements in GAN training on smaller datasets, adds up to a complete plan for reliable and efficient use of fNIRS data in the creation and assessment of advanced machine learning models.

The main contribution of the current work is to classify different brain states with higher classification accuracy with the help of GAN. In doing so, this work aims to investigate the effects of stress on the patterns of spatial activation elicited by the challenging Stroop color–word task (SCWT). In addition, the current study aims to improve the brain state using binaural beat stimulation. We conducted morning and evening sessions with working individuals in anticipation of the participants experiencing stress due to the demands of the workday. Our main objective is to examine how participants’ hemodynamic responses to workplace stress change over time, determine whether these changes can be reliably detected using classification techniques, and determine how stress levels are reduced when exposed to binaural beats.

## 2. Materials and Methods

### 2.1. Participants

Twenty-one healthy volunteers—thirteen men and eight women—working at the American University of Sharjah participated in this study (mean age: 29 ± 5 years). The subjects’ vision was either normal or corrected to normal. None of the participants reported having issues with hearing or seeing color. There was no history of neurological or visual impairment in any of the participants, and there was no proof of drug addiction or ongoing medication use. On the day of the experiment, the participants were instructed not to consume any alcohol, caffeine, or other drinks that would increase their energy levels. Prior to starting the experiment, every participant was given a comprehensive explanation about the study and every participant had the choice to withdraw from the experiment at any moment. Written consent forms were signed prior to the experiment. The American University of Sharjah’s Institutional Review Board granted permission for this study, which was carried out in compliance with the most recent Helsinki Declaration [30].

### 2.2. Task Design

This study used the SCWT as a stress-inducing paradigm. As part of the assignment, participants had to pay attention to six distinct color words that were presented in random order: “Green”, “Yellow”, “Red”, “Cyan”, “Magenta”, and “Blue”. Notably, the word displayed on the computer screen was printed in a color that was not consistent with its semantic meaning. A cognitive challenge was introduced by asking the participants to select the color of the typed word as a response, not the word itself. Participants had to select the ink color from six options that were displayed as push buttons underneath the displayed word. The background of the buttons featured a third color, and the text on the push buttons appeared in a different color to increase the complexity of the task. Rather than identifying the color of the button itself, participants were asked to identify the color written inside it.

**Figure 1:**
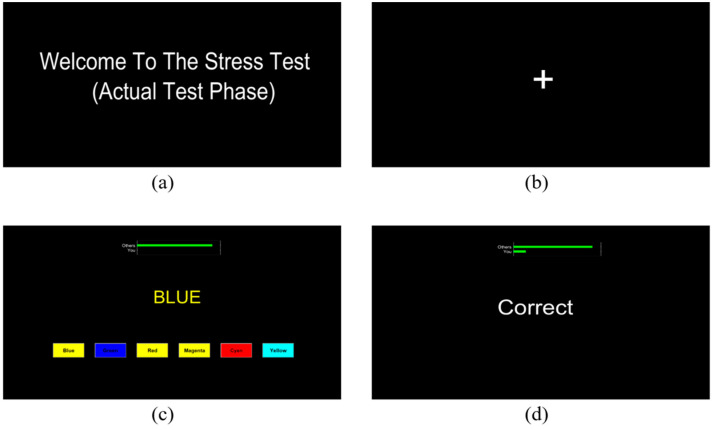
An example of Stroop color–word task used as a stressor in the current study: (**a**) Welcome screen (**b**) Rest phase (**c**) The SCWT task in which the participant had to choose the word yellow (font color of the displayed word, i.e., BLUE) written in the cyan color box. (**d**) Feedback on choosing the right option.

Every question had a time limit, and if the participant did not answer in the given amount of time, a “Time is out” notification was displayed on the screen. Participants also received feedback about how correct their selected option was on the screen. MATLAB^®^ 2023 was used to implement the SCWT protocol, guaranteeing accuracy and consistency in task execution. Figure 1 illustrates an example of a question that participants saw on their screens.

### 2.3. Experimental Paradigm

**Figure 2:**
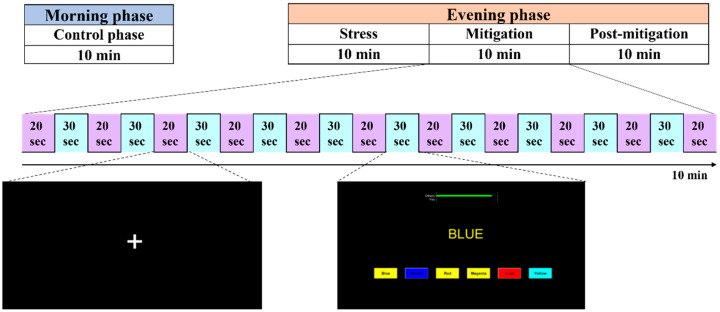
Experimental paradigm.

The experiments were carried out at two different times of the day, i.e., morning and afternoon. There were 10 trials in a session of the experiment, each lasting 50 s (30 s task period). One experimental session, known as the control phase, was conducted in the morning, whereas three sessions were conducted in the afternoon, namely stress, mitigation, and post-mitigation. During the afternoon experiments, participants carried out the experiments in the stress phase (first phase) in a manner similar to that of the control phase. Afterward, during the mitigation phase (second phase), participants performed the SCWT tasks while simultaneously being stimulated by binaural beats. Ultimately, the third session (post-mitigation) replicated the stress or control phase by having participants complete multiple SCWT trials. A summary of the experimental paradigm is given in Figure 2.

Participants sat in a comfortable chair and were instructed to move their bodies as little as possible during the experiment. For all stimulation durations, a 20-s inter-stimulation interval was kept, along with 20-s pre- and post-rest intervals. During the rest period, a black screen was presented. Visual stimuli were displayed to the subjects on a computer screen, and they were instructed to keep their eyes open throughout the experiment.

### 2.4. Optode Placement

**Figure 3:**
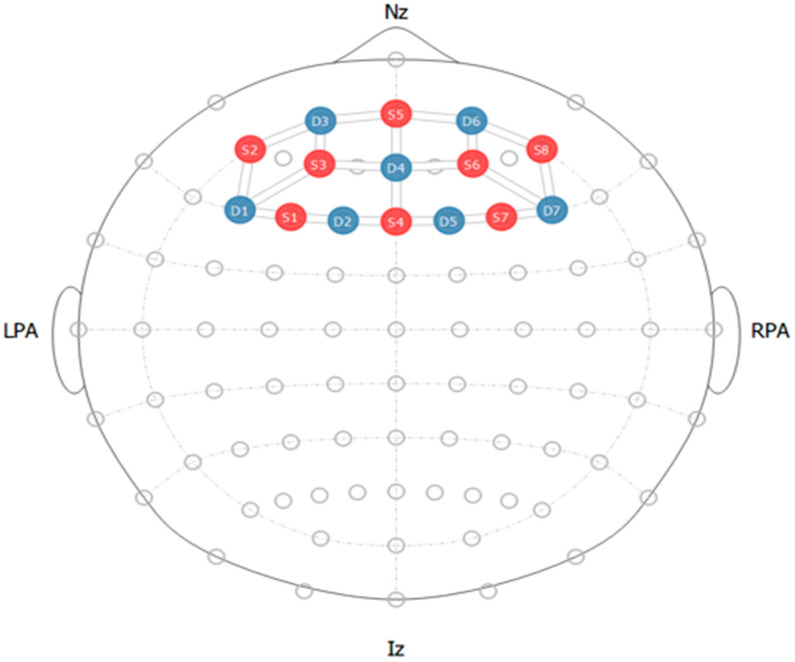
fNIRS optode configuration with red dots showing sources and blue showing detectors.

In order to record brain signals, the prefrontal cortex region was covered with seven detectors and eight emitters. The optode configuration on the region of interest is displayed in Figure 3. The Fpz region of the brain was chosen as the reference point to guarantee exact placement on the prefrontal cortex. This reference point was chosen using the International 10–20 System for electrode placement to guarantee correct electrode placement.

### 2.5. Data Acquisition

Brain signals was sampled at a frequency of 10.17 Hz for this study. A single-phase continuous wave fNIRS system, NIRSport2 from NIRx Medical Technologies, Orlando, FL, USA, was used to acquire the fNIRS signals. Two different wavelengths were used by the system: 760 nm and 850 nm. The process of converting raw intensities into changes in ΔHbO and ΔHbR was carried out by utilizing the Modified Beer-Lambert Law [31]. *NIRSlab* was used to convert the data from the light intensity to the changes in hemoglobin.

### 2.6. fNIRS Preprocessing

Several pipelines have been employed by researchers to preprocess the fNIRS data [32]. After the acquisition, the data (ΔHbO & ΔHbR) for the current study were preprocessed to remove any noise contamination that could have impacted the signal quality. In order to correct for artifacts linked with subjects’ movement, the converted ΔHbO and ΔHbR intensities were first subjected to principal component analysis followed by temporal derivative distribution repair [33,34]. Following motion-artifact correction, a Butterworth bandpass filter with a low-pass cutoff frequency of 0.15 Hz and a high-pass cutoff frequency of 0.01 Hz was applied to remove cardiac, respiratory, and low-frequency drift signals. Lastly, the desired hemodynamic response function (dHRF) is utilized in this work to identify neuronal activation. Two gamma functions were applied to produce the dHRF, as explained by [35].

### 2.7. Statistical Analysis

The mean of ΔHbO, *t*-values, and *p*-values were utilized in the study for statistical analysis and the identification of active channels. The degree of freedom of the trial period was used to choose the *t*_crt_, and a significance level for the one-tailed *t*-test was set at 0.05. In order to calculate the *t*-values, MATLAB^®^’s built-in *robustfit* function was utilized. A channel was considered active when the *t*-value was greater than *t*_crt,_ and the *p*-value was less than 0.05.

### 2.8. Data Augmentation

**Figure 4:**
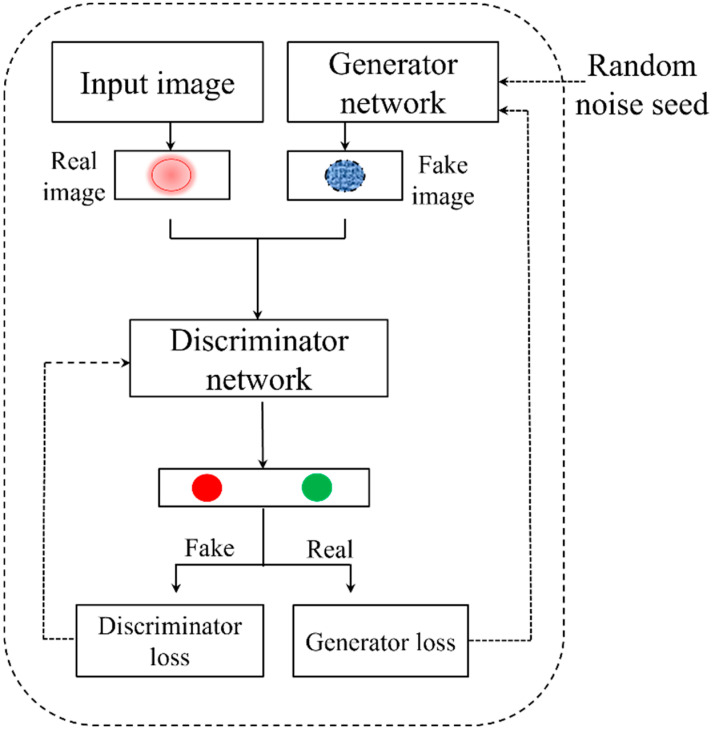
General architecture of generative adversarial network.

For data convolution in this study, we used a deep convolutional generative adversarial network (DCGAN) [36]. GANs were initially introduced for image generation. We first explain their basic idea with reference to image generation. We follow this up with the adaptation to our use case. A GAN consists of a discriminator and a generator network. The generator learns the distribution of the data and creates images from random noise inputs. Concurrently, the discriminator assesses whether an image is deemed “real” and gives losses for generator and discriminator networks [37]. Figure 4 shows the general architecture of GAN.

On the other hand, the DCGAN algorithm combines GAN with convolutional neural networks (CNN). With a discriminator network and a generator network, the DCGAN basic architecture is similar to that of GANs. The discriminator network uses convolutional and normalization layers, capped by a dense layer, to evaluate the authenticity of images, whereas the generator network uses transposed convolutional and normalization layers to convert a random noise vector into images. In the competitive training dynamic, the generator wants to produce images that are realistic, while the discriminator wants to correctly identify generated images as fraudulent [38].

The discriminator in DCGANs consists of a sequence of convolution layers with stride convolutions, each of which down-samples the input image. With each layer, the network learns the complex representation of input images for classification (fake or real). The discriminator loss is given by

$$D_{r}=\text{log}\;(D(x))$$

$$D_{f}=\text{log}\;(1\;-\;D(G(z)))$$

where x is the input data, z is the noise vector, D(*x*) is the output of the discriminator for the real image, and D(G(*z*)) is the output for the fake image. The main goal of the discriminator is to minimize D(G(*z*)) and maximize D(*x*). The overall loss of discriminator is given by

$$D_{l}=\frac{1}{m}\sum_{i=1}^{m}({\;\text{log}\;(D(x}^{i}))+\text{log}\;(1\;-{\;D(G(z}^{i}))))\;$$

In contrast, the generator comprises convolution layers that use fractional-strided convolutions or transpose convolutions, resulting in up-sampling of the input picture at each convolutional layer. As the noise moves through the layers, the network gradually increases the image size to match that of a real image. The loss function for the generator is defined as

G=log (1 − D(G(z)))

$$G_{l}=\frac{1}{m}\sum_{i=1}^{m}\;\text{log}\;(1\;-{\;D(G(z}^{i})))$$

where the aim is to maximize D(G(z)). Moreover, the discriminator and the generator are continuously optimizing themselves, which can be represented as follows:

$$\min_{G}\max_{D}V\left(D,G\right)=E_{x\sim p_{data}\left(x\right)}\left[log\;D\left(x\right)\right]+E_{z\sim p_{z}\left(z\right)}\left[\log\left(1-D\left(G\left(z\right)\right)\right)\right]$$

where $p_{z}\left(z\right)$ is the input noise, E represents the expectation. Table 1 summarizes the main parameters of the DCGAN. The activation function used was the *LeakyReLU* function.

### 2.9. Classification

After processing the data, the next task was to classify the data using two different methods: classification based on temporal features and classification using images. For the image-based classification, five different classifier types—feed-forward NN (FFNN), linear support vector machines (LSVM), decision tree (DT), restricted Boltzmann machine (RBM), and CNN—were employed. On the other hand, linear discriminant analysis (LDA) was used for the classification based on temporal features. The mean, maximum (max), slope, skewness (skew), and kurtosis (kurt) were among the temporal features that were extracted for four distinct window sizes of 0 to 5 s, 5 to 30 s, 30 to 50 s, and 0 to 50 s. The classification accuracy was computed using fivefold cross-validation.

The data from all trials were first converted into visual representations for image-based analysis. In the current study, activation maps and connectivity maps were used as image features. Activation maps were produced using a *t*-test, and MATLAB’s robustfit function was used to calculate the *t*-values for each channel. Each trial was further divided into a 5-s window (resulting in the generation of 10 images from each trial) in order to increase the size of the data. Functional connectivity (FC) in the brain refers to the interactions between different brain regions, characterized by the temporal correlations observed between neurophysiological activities in spatially separated areas. These interactions are represented on the connectome, which maps individual differences in brain organization and highlights the potential of connectivity-based approaches for biometric applications. In this study, Pearson’s correlation coefficients (*r*) were calculated using temporal data from all channels to create connectivity matrices. These matrices detail both intra- and inter-hemispheric connectivity. The matrix elements are the correlation coefficients between paired channels, with the rows and columns corresponding to the channel numbers. Once the activation maps and FC maps were acquired, CNN, FFNN, LSVM, DT, and RBM were the classification methods used for these images. Again, fivefold cross-validation was used to obtain the average classification accuracy.

## 3. Results

### 3.1. Comparison of Hemodynamic Responses

**Figure 5:**
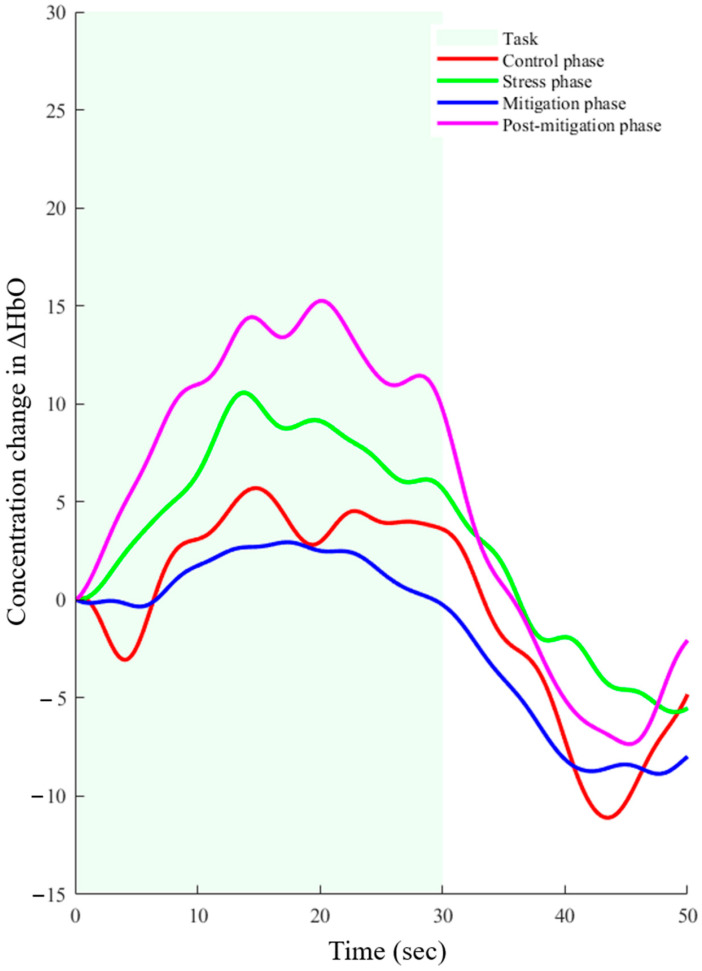
Averaged hemodynamic response achieved for all four phases.

The hemodynamic response function (HRF) is a common pattern for any type of activation seen in the human brain. The derived hemodynamic response function (dHRF) was utilized to identify the trials that exhibited activation to characterize the shape of the HRF. After extraction, the active trials were averaged for each subject. The control phase, stress phase, mitigation phase, and post-mitigation phase were all processed using the same procedure. In each of the four cases, visual inspection verified credible activation. However, there was a discernible difference between the hemodynamic response seen prior to and following the binaural beats sessions (*p*-value < 0.05). The averaged hemodynamic response activation patterns for each of the four cases are shown in Figure 5. The task period is indicated by the green shaded area, and the rest interval is indicated by the non-shaded area.

*T*-tests were used to determine the statistical significance of the activation level in each of the four cases. Paired *t*-tests were used to compare activation in stress, mitigation, and post-mitigation sessions, and independent sample *t*-tests were used to compare the average activation in the control phase with the other three cases. A difference met both of the following requirements to be deemed statistically significant: (i) *p*-value < 0.05 and (ii) *t*-value > critical *t*-value (t_crt_).

A statistically significant difference was noted in the mean activation levels between the control and stress phases (*p*-value < 0.001). Interestingly, during the binaural beats session, there was no statistically significant difference in the responses between the mitigation phase and control phase (*p*-value = 0.377). However, a significant difference was found when stress phase data were compared with post-mitigation phase data (*p*-value < 0.005). The activation of the brain after the binaural beats session (post-mitigation) was significantly higher (*p*-value < 0.005) than it was during the control phase. Overall, a significant improvement was noticed in the hemodynamic response because of the binaural beat stimulation.

### 3.2. Enhancement of Classification Accuracies

**Figure 6:**
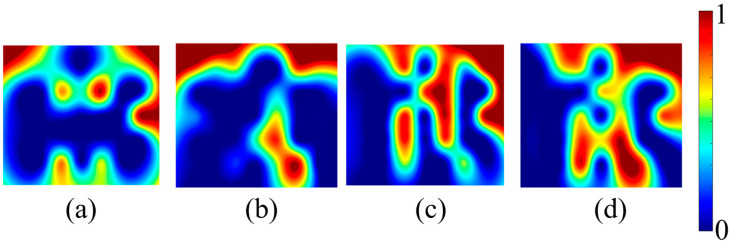
Averaged activation maps for each of the four phases: (**a**) control, (**b**) stress, (**c**) mitigation, and (**d**) post-mitigation.

**Figure 7:**
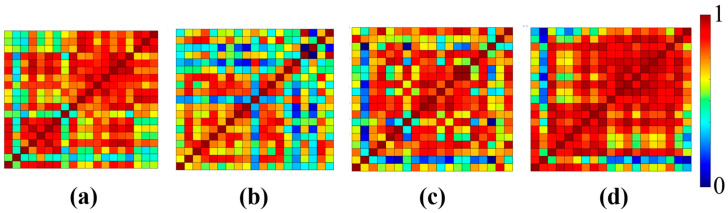
Averaged connectivity maps for each of the four phases: (**a**) control, (**b**) stress, (**c**) mitigation, and (**d**) post-mitigation.

Figure 6 and Figure 7 show the averaged activation maps and connectivity maps, respectively. As mentioned earlier, there were two different methods used in the classification process: the first was the use of temporal features, and the second was the use of brain maps as features. In the case of classifying using time series data, all temporal features were extracted and subsequently utilized in pairs. The LDA-based classification was performed for each of the four feature extraction windows. Table 2 shows the classification accuracy obtained with each of the feature sets.

Given the two-class classification, the highest achieved classification accuracy in all comparisons was 60.69%, which could be regarded as a moderate result. Furthermore, no statistically significant difference was found in the classification accuracies when comparing the three different cases, i.e., control vs. stress, control vs. mitigation, and control vs. post-mitigation. Brain activation maps and connectivity maps are widely utilized in both fMRI and fNIRS to find the activated areas. These maps served as features in the classification process for the current study. For a single 50-s trial, ten activation maps and ten connectivity maps were produced having five seconds of data each. This procedure produced a total of 100 brain activation map images and 100 connectivity map images (10 trials each, resulting in 10 maps) for a single subject. A total of 2100 images were collected from 21 subjects for each of the four scenarios (control, stress, mitigation, and post-mitigation). From this dataset of 2100 images per class, five different classifiers—FFNN, LSVM, DT, RBM, and CNN—were used to perform classification similar to the classification based on temporal features. Table 3 shows the resulting classification accuracies obtained using the brain activation maps. Similarly, Table 4 shows the obtained classification accuracies using the connectivity maps.

In this case the maximum classification accuracy achieved was 73% for Control vs. post-mitigation. Lastly, the acquired 2100 images per class were used as an input data set to augment the dataset using DCGAN. After almost doubling the size of the data, the augmented dataset was used to perform classification using the four classifiers mentioned earlier. The classification accuracy obtained with the augmented brain activation maps is shown in Table 5. Similarly, Table 6 shows the obtained classification accuracies obtained using augmented connectivity maps.

## 4. Discussion

The purpose of this work was to investigate whether DCGAN can be used to augment the data based on hemodynamic responses (i.e., ΔHbO) in the brain’s frontal cortex that could improve classification/detection accuracy of brain state. SCWT was utilized to activate specific brain activity during the acquisition of fNIRS signals across four distinct brain states: control, stress, mitigation, and post-mitigation. Initially, temporal features were utilized in pairs to facilitate the initial implementation of LDA-based classification.

Next, using brain activation maps as a feature, five classifiers were used for the classification process: CNN, FFNN, LSVM, DT, and RBM. Lastly, DCGAN was used for data augmentation, which aided in improving classification accuracy in stress detection and confirmed the efficacy of the suggested technique.

The primary objective of this study is to contribute to the advancement of classification accuracy, particularly in detecting specific brain states such as stress. One of the main objectives of fNIRS-based brain imaging is to identify abnormal brain conditions, such as stress, anxiety, dementia, Alzheimer’s, mild cognitive impairment, etc. The requirement for automated procedures in accurately detecting these mental states is of importance, and achieving higher classification accuracies leads to robust and reliable outcomes.

During the experiment, the performance of SCWT under time constraints, in addition to the feedback they received, significantly increased the stress levels in participants. This was reflected by noticeable changes in the participants’ behavior, as they showed signs of increased anxiety and nervousness during the SCWT task. As the study participants were all employed individuals, the hypothesis was that the brain states in the morning (control) and afternoon (stress, mitigation, and post-mitigation) would differ significantly from one another, as shown in Figure 5. Statistical analysis of the hemodynamic response during the stress and mitigation phases showed no significant difference. Nonetheless, a statistically significant difference (*p*-value < 0.001) was found in the hemodynamic response for the control and post-mitigation phases. Furthermore, there was a statistically significant difference in hemodynamic response between the post-mitigation and stress phases, indicating that the brain state was enhanced by binaural beat stimulation. In Figure 5, it can be noticed that the activation levels during the stress and post-mitigation phases are higher as compared with the control phase. This can be due to adaptation of the task.

For the classification based on temporal features, a feature set comprising five features was extracted using the hemodynamic responses in each of the four groups. Four different window sizes were used to extract these features: 0–5 s, 5–30 s, 30–50 s, and 0–50 s. The objective of using the extracted features in pairs was to distinguish the control state from the other three brain states—stress, mitigation, and post-mitigation. LDA was employed for this classification, given its widespread use as a classifier for temporal fNIRS data [39]. There was no significant difference obtained between the classification accuracies of the three cases that were examined. This finding showed the limitation of relying solely on temporal features for the detection of specific brain states, highlighting its infeasibility as a viable option.

The prefrontal cortex plays an important role in various active-memory tasks, including mental arithmetic, mental counting, and working-memory tasks, and is predominantly responsive to mental training. As people age, the functional capacity of this brain area weakens, potentially leading to devastating conditions like Alzheimer’s. The variations in mental health associated with such changes can be effectively followed through brain activation maps. Therefore, the impact of binaural beats was also verified by examining the changes in activation maps.

Each trial of SCWT was divided into 5 s blocks, which were then used to make the activation maps and connectivity maps of the prefrontal cortex area of the brain. Since every trial lasted for 50 s, 10 images were generated from each trial. These maps were used to perform classification using five different classifiers: CNN, FFNN, LSVM, DT, and RBM. Out of the five classifiers, FFNN provided the lowest accuracy (52%). The statistical findings derived from examining ΔHbO signals were supported by the classification accuracy of each of the five classifiers. All the classifiers demonstrated the highest classification accuracy in the control vs. post-mitigation scenario, corresponding to a statistically significant difference in hemodynamic responses between the two groups. Additionally, CNN produced the highest classification accuracy, outperforming all other classifiers. Moreover, the accuracy that was obtained was about 70%.

Lastly, DCGAN was used to augment the images of brain activations maps and connectivity maps, and the five classifiers mentioned earlier were used to classify the images. A significant increase in the classification accuracies was noticed when the augmented data were used for classification (shown in Table 3). By using augmented data, the previously achieved classification accuracy of 73% using CNN increased to 94%. CNN gave the best classification accuracies, which were 91%, 86%, and 94% for control vs. stress, control vs. mitigation, and control vs. post-mitigation, respectively. Similarly, by utilizing the augmented connectivity maps, the classification accuracy went up to 96%. DCGAN significantly improved the classification accuracy for fNIRS-based imaging. Through utilizing the proposed method, the detection of any abnormalities in brain states can be detected efficiently.

The scope of the study was limited to prefrontal cortex only due to the small number of fNIRS channels that were available. By adding more channels to explore different brain regions, future studies can expand their scope of investigation. Moreover, the combination of hybrid EEG-fNIRS neuroimaging modalities presents a promising way to obtain brain signals, enabling whole-brain examination and verifying the study’s hypotheses [40,41]. It is necessary to acknowledge a primary limitation of fNIRS—inter-subject and intra-subject variability in the hemodynamic response signal [42]. This research overcame this constraint by combining information from several participants, which enabled a thorough evaluation of the overall trend. Subsequent studies could examine optode placements at high densities or in bundles, with short separation channels included, to enhance spatial resolution and boost activation map accuracy [9]. Future research may examine neuroplasticity in the hours or days following binaural beats therapy to determine the long-lasting effects. Future studies will focus on systematically exploring and comparing different data augmentation techniques, as well as investigating the impact of varying DCGAN parameters to further enhance the robustness and generalizability of fNIRS-based classification models. A further constraint concerns the study’s sample size, which, although comparable to earlier research, implies the possibility of growing to a larger cohort. Furthermore, gender variability was not investigated in this study; one possible direction is to perform independent research on male and female participants for comparative analysis. In addition, future studies might concentrate on applying electrical or magnetic stimulation only to particular brain regions in order to evaluate their varying effects on different brain regions [43,44].

## 5. Conclusions

In conclusion, this study tackles the challenge of detecting distinct brain states with higher accuracy, having an emphasis on the impact of stress during the Stroop color–word task through functional near-infrared spectroscopy (fNIRS). Using a variety of classification approaches, we encountered the frequently occurring constraint of limited participant numbers which leads to lower accuracy. The introduction of data augmentation through a deep convolutional generative adversarial network proved critical in increasing classification accuracy to 96%. Notably, the same classification approaches fell short with the original data set (without augmentation), highlighting the importance of data augmentation in improving interpretability. The investigation of binaural beat stimulation adds a stimulating element to our study, implying possible stress reduction benefits. The results demonstrate the potential of fNIRS in detecting subtle changes in brain states, especially when combined with bigger datasets obtained through data augmentation. This study adds vital insights to the advancement of brain state identification and opens up a prospective route for advanced interpretations in the field of functional brain imaging.

## Figures and Tables

**Table 1:** Main Parameters of DCGAN.

Parameters	Values
Batch size	32
Epoch	30,000
Learning rate	0.0001
Image Channels	3
Batch Normalization	0.9
Drop-Out	0.3
Strides	2

**Table 2:** LDA-based classification accuracies (%) obtained for four different window sizes.

Window Size	Control vs. Stress	Control vs. Mitigation	Control vs. Post-Mitigation
0 to 5 s	58.84	59.06	59.04
5 to 30 s	59.53	59.60	60.04
30 to 50 s	58.58	59.50	59.29
0 to 50 s	59.56	59.81	59.96

**Table 3:** Classification accuracies obtained using activation (not augmented). Classifiers used: feed-forward neural network (FFNN), linear support vector machine (LSVM), decision tree (DT), restricted Boltzmann machine (RBM), and convolutional neural network (CNN).

Accuracy (%)			
Classifiers	Control vs. Stress	Control vs. Mitigation	Control vs. Post-Mitigation
CNN	72	68	73
FFNN	57	52	59
DT	64	60	63
LSVM	66	63	67
RBM	62	59	64

**Table 4:** Classification accuracies obtained using connectivity maps (not augmented). Classifiers used: feed-forward neural network (FFNN), linear support vector machine (LSVM), decision tree (DT), restricted Boltzmann machine (RBM), and convolutional neural network (CNN).

Accuracy (%)			
Classifiers	Control vs. Stress	Control vs. Mitigation	Control vs. Post-Mitigation
CNN	76	64	73
FFNN	61	52	65
DT	61	56	64
LSVM	68	64	69
RBM	63	56	66

**Table 5:** Classification accuracies obtained using augmented brain activation maps. Classifiers used: feed-forward neural network (FFNN), linear support vector machine (LSVM), decision tree (DT), restricted Boltzmann machine (RBM), and convolutional neural network (CNN).

Accuracy (%)			
Classifiers	Control vs. Stress	Control vs. Mitigation	Control vs. Post-Mitigation
CNN	91	86	94
FFNN	62	57	64
DT	88	85	89
LSVM	84	80	86
RBM	76	70	75

**Table 6:** Classification accuracies obtained using augmented connectivity maps. Classifiers used: feed-forward neural network (FFNN), linear support vector machine (LSVM), decision tree (DT), restricted Boltzmann machine (RBM), and convolutional neural network (CNN).

Accuracy (%)			
Classifiers	Control vs. Stress	Control vs. Mitigation	Control vs. Post-Mitigation
CNN	93	88	96
FFNN	66	61	70
DT	91	87	94
LSVM	85	82	86
RBM	74	71	77

## Data Availability

Data underlying the results presented in this paper are not publicly available at this time but may be obtained from the authors upon reasonable request.
